# Isolated low high density lipoprotein-cholesterol (HDL-C): implications of global risk reduction. Case report and systematic scientific review

**DOI:** 10.1186/1475-2840-4-1

**Published:** 2005-01-04

**Authors:** Melvin R Hayden, Suresh C Tyagi

**Affiliations:** 1 Department of Family and Community Medicine, University of Missouri Columbia, Missouri, PO BOX 1140 Lk. Rd. 5-87, Camdenton, Missouri 65020 USA; 2 Department of Physiology and Biophysics, 500 South Preston Street, University of Louisville, Louisville, Kentucky 40292 USA

**Keywords:** Apo A-1, ABCA1, Atheroscleropathy, Atherosclerosis, antioxidant, anti-inflammatory, lipoprotein A, redox stress, fibrates, niacin, ezetimibe, statins.

## Abstract

**Background:**

The importance of low high-density lipoprotein cholesterol (HDL-C), elevated non HDL-C (as part of the metabolic syndrome, prediabetes, and type 2 diabetes mellitus), and an isolated low HDL-C is rapidly emerging. The antiatherosclerotic roles of reverse cholesterol transport and the pleiotropic antioxidant – anti-inflammatory mechanistic effects of HDL-C are undergoing rapid exponential growth.

**Case presentation:**

In 1997 a 53-year-old Caucasian male presented with a lipoprotein profile of many years duration with an isolated low HDL-C and uric acid levels in the upper quintile of normal. He developed an acute myocardial infarction involving the right coronary artery and had percutaneous transluminal coronary angioplasty with stenting of this lesion. He also demonstrated a non-critical non-flow limiting lesion of the proximal left anterior descending coronary artery at the time of this evaluation.

Following a program of global risk reduction this patient has done well over the past 7 years and remains free of any clinical signs and symptoms of atherosclerosis. His HDL-C and uric acid levels are currently in the normal physiological range.

**Conclusion:**

Low HDL-C and isolated low HDL-C constitute an important risk factor for atherosclerosis. Therapies that lead to a return to normal physiologic range of HDL-C may result in the delay of atherosclerotic progression.

## Case presentation

MRH, a 53-year-old Caucasian male (physician) developed an acute inferior myocardial infarction (MI) associated with bradycardia and occasional PVCs. Emergency medication included aspirin, nitroglycerin and a bolus of TPA.

The cardiology team preformed PCTA at the site of near complete blockage of the right coronary artery with successful stent placement. At this time a non-critical 40% lesion located in the proximal left anterior descending coronary artery was noted, which was not manipulated. The patient was discharged following 24 hours of stable monitoring.

### Past Medical History

Relapsing fever 1971 full recovery, spontaneous left pneumothorax times two (1982–83), lumbar fusion back surgery 1985, and *Herpes Simplex *encephalitis 1989 with full recovery.

### Family History

Mother with CVA (cerebellar) age 58 full recovery. Died of Hodgkin's lymphoma 64. Brother with type 1 diabetes mellitus with onset at age 29 (known PAD and aorto-femoral bypass age 49) died in sleep age 51.

Father with CVA (vertebrobasilar) age 75 with full recovery, COPD, died in sleep while recovering from TIA and pneumonia age 84.

Grandparents lived to their 80s and died of old age.

### Social History

High stress family physician who seldom drank alcohol and smoked a pipe occasionally. Blood pressure at times of high stress would elevate to 140/85–88 and return to 120–130s/ 70–75 at times of non-stress in the office. He was physically active with no dedicated exercise program

### Laboratory Values

Five months prior to MI and reflective of numerous metabolic profiles over the preceding decades.

Total cholesterol 198 mg/dL

Triglycerides 154 mg/dL

HDL-C 34 mg/dL. HDL-C (1970–1973 32 mg/dL and 34 mg/dL)

LDL-C calculated 120 mg/dL

Non HDL-C = (198-34) = 164

Total Chol/HDL ratio = 6.2 > than 5 and is high

Uric acid 6.5 mg/dL

Blood sugar non-fasting 102 mg/dL

Homocysteine first week post MI fasting: 28 mcmol/L

LFTs, electrolytes, calcium and phosphorus, serum iron, renal function, and CBC were all in normal range.

Patient started a program reflecting the global risk reduction approach described in the RAAS acronym (table 1) and is currently taking an angiotensin receptor blocker, aspirin, beta blocker, folic acid, and a statin. Patient was intolerant of ACE inhibitor therapy due to cough and fatigue and has been unable to tolerate niacin on numerous attempts both pre and post MI due to incapacitating headaches.

**Table 1:** The RAAS acronym: global risk reduction

***R***	**Reductase inhibitors (HMG-CoA). **Decreasing modified LDL-cholesterol, i.e. oxidized, acetylated LDL-cholesterol. Decreasing triglycerides and increasing HDL-cholesterol Improving endothelial cell dysfunction. Restoring the abnormal Lipoprotein fractions. Thus, decreasing the redox and oxidative stress to the arterial vessel wall and myocardium.
**Redox stress reduction.**	
**A**	**AngII inhibition or blockade:**
	**ACEi-prils. ARBS-sartans. **Both inhibiting the effect of angiotensin-II locally as well as systemically. Affecting hemodynamic stress through their antihypertensive effect as well as the deleterious effects of angiotensin II on cells at the local level – injurious stimuli -decreasing the stimulus for O_2_. production. Decreasing the A-FLIGHT toxicities. Plus the direct-indirect antioxidant effect within the arterial vessel wall and capillary. Antioxidant effects. Aspirin antiplatelet, anti-inflammatory effect. Adrenergic (non-selective blockade) in addition to its blockade of Prorenin→Renin Amlodipine with its calcium channel blocking antihypertensive effect, in addition to its direct antioxidant effects.
**Redox stress reduction.**	
**A**	**Aggressive control of diabetes **to HbA_1c _of less than 7. (This usually requires combination therapy with the use of: Insulin secretagogues, insulin sensitizers (thiazolidinediones), biguanides, alpha-glucosidase inhibitors, and ultimately exogenous insulin.) Decreasing modified LDL cholesterol, i.e. glycated – glycoxidated LDL cholesterol. Improving endothelial cell dysfunction. Also decreasing glucotoxicity and the oxidative – redox stress to the intima and pancreatic islet. **Aggressive control of blood pressure**, which usually requires combination therapy, including thiazide diuretics to attain JNC 7 guidelines. **Aggressive control of dyslipidemias**, which frequently requires combination therapy (especially in the metabolic syndrome and T2DM), including TLC, statins, fibrates, selective cholesterol inhibitors such as ezetimibe, and niacin **Aggressive control of Hcy **with folic acid with its associated additional positive effect on re-coupling the eNOS reaction by restoring the activity of the BH4 cofactor to run the eNOS reaction and once again produce eNO.
**Redox stress reduction.**	
**S**	**Statins. **Improving plaque stability (pleiotropic effects) independent of cholesterol lowering. Improving endothelial cell dysfunction. Plus, the direct – indirect antioxidant anti-inflammatory effects within the islet and the arterial vessel wall promoting stabilization of the unstable, vulnerable islet and the arterial vessel wall. Style: Lifestyle modification: lose weight, exercise, and change eating habits. Stop Smoking
**Redox stress reduction**	

Current Laboratory Values 2004:

Total cholesterol: 138 mg/dL

Triglycerides: 94 mg/dL

HDL-C: 45 mg/dL

LDL-C calculated: 74 mg/dL

Non HDL-C: (138-45) = 93

Total Chol/HDL ratio = 3.0

Uric acid: 6.5 mg/dL

Blood sugar: Fasting 80 mg/dL, 2 hour post prandial 118 mg/dL

Homocysteine: 7.2 mcmol/L

Lp(a): 4.2 mg/dL in normal range immediate post MI and again at this time: 4.3 mg/dL.

hs-CRP: 0.7 mg/L.

LFTs, electrolytes, calcium and phosphorus, serum iron, renal function, and CBC are all in normal range.

This patient has done well over the past seven years and remains free of any clinical signs and symptoms of cardiovascular disease. While this patient will always remain a CHD risk, his current laboratory values remain in a normal physiological range. As noted above his HDL-C and uric acid levels are currently in the normal physiological range and his hs-CRP remains in the second quartile.

#### Comment

According to Framingham risk scores associated with the National Cholesterol Education Program Adult Treatment Panel III (NCEP ATP III) guidelines [1], few would have recommended any treatments other than therapeutic lifestyle changes (TLC) and possibly niacin, which our patient was intolerant both pre and post event in 1997.

If we score this patient according to the estimate of 10-year risk for men he gets 6 points for age 53, 2 points for total cholesterol 160–199 age 53, 3 points for being a pipe smoker, 2 points for HDL being < 40 mg/dL, and 1 point for systolic blood pressure 140–159 untreated. This totals 14 points and results in an estimated 10-year risk for men of 16%, which is less than the 20% recommended for more aggressive therapy.

Even if we apply the NCEP ATP III guidelines of having two plus risk factors: Male sex, hypertension, smoking, and low HDL-C with a 10 risk < or = to 20% we obtain the following recommendations: LDL-C goal < 130 mg/dL, initiation of TLC if LDL-C is = or > 130 mg/dL, consideration of drug therapy if LDL-C is > or = to 130 mg/dL after three months of TLC. It is important to note that our patient had a LDL-C of 120 mg/dL prior to his event. Even if we look at the non HDL-C levels, which are allowed to be 30 mg/dL higher than LDL-C goals we have a patient with a non HDL-C of only 164. MRH became a CHD risk patient within a short period of time of 5 months.

## Discussion

The importance of low HDL-C and cardiovascular disease associated with the lipid triad (Low HDL-C, elevated triglycerides, and increased small dense LDL-C) found in the metabolic syndrome (metS) and overt type 2 diabetes mellitus (T2DM) and a contributing factor to the elevated non HDL-C discussed in the current NCEP ATP III guidelines or the patients with isolated low HDL-C is rapidly evolving.

The accelerated atherosclerosis (atheroscleropathy) associated with the metS and T2DM has been previously reviewed and is definitely a serious problem associated with the current epidemic of obesity – diabesity and T2DM [2-4].

Both isolated low HDL-C and elevated non HDL-C (total cholesterol minus HDL-C) levels are difficult to get to known NCEP ATP III recommendations and this task usually requires combination therapy. These therapies consist of therapeutic life style changes and pharmacotherapy including statins, fibrates, selective cholesterol inhibitors such as ezetimibe, and niacin in addition to a global risk reduction of all non HDL-C existing risk factors (table 2) [5].

**Table 2:** Effects of drugs on HDL-C levels

**DRUG**	**PERCENT INCRESE**
Nicotinic acid (niacin)	15% – 35%
Fibrates	10% – 15%
Estrogens	10% – 15%
Statins Coupled Dual Effect Associated with potent LDL-C reduction, which make the statins "shine"	5% – 10%
Alpha blockers	10% – 20%
Alcohol (in moderation)	10%
Ezetimibe	3%

In this case report a focus on isolated low HDL-C is appropriate. This case report demonstrates a marked improvement of all lipid parameters including his low HDL-C. However, this marked improvement is not always as simple as this case and therefore, both the patient and the clinician need to be very patient, as well as, creative in order to achieve global risk reduction [5].

### Isolated low HDL-C

In 1977 the Tromso Heart Study demonstrated that CAD patients have HDL-C levels 35% lower than controls and those patients with low HDL-C are three times more likely to develop CAD than those with elevated LDL-C [6]. These early views certainly support the concept that an isolated low HDL-C is a common antecedent of clinical CHD, as well as being important in accelerating the progression of atherosclerosis.

The inverse relation of HDL-C to CHD events has been widely discussed since the original publication of data from the Framingham study (1986) [7,8]. Castelli WP *et al. *were able to show an inverse association of high HDL-C and low coronary risk was as statically as strong as the direct association of high LDL-C and high coronary risk in a cohort of men and women age 40–82 followed for 12 years who were free from CAD at study entry. At any level of cholesterol low HDL-C increases the rate of CHD [1]

The NCEP ATP III guidelines clearly defines a level < 40 mg/dL as an independent risk factor for CHD [1]. Raising HDL-C is not a target for either primary or secondary prevention at this time, however its importance as a tertiary target is rapidly emerging.

Michael Miller has stated: "Low HDL-C is the most common lipoprotein abnormality in patients with CHD and is predictive of CHD events, even when total cholesterol levels are normal" [9].

Goldbourt U *et al.*, found that the prevalence of isolated low HDL-C as a risk factor for CHD mortality to be present in one out of six or 16.6 % while studying a 21-year follow up of 8000 men [10]. Furthermore, they found that an excess CHD risk associated with isolated low HDL-C appeared particularly increased in men with diabetes mellitus, whose death rate was 65% higher than in diabetics with HDL-C > 0.9 mmol/L or 36 mg/dL.

There are at least eight secondary causes for low HDL-C (table 3) and at least seven drugs that have a positive effect on raising HDL-C (table 2). As demonstrated in our case report, the beneficial effects of raising HDL-C with statin therapy and a program of global risk reduction have been positive in preventing the progression of atherosclerosis and recurrent acute coronary syndromes (table 4).

**Table 3:** Secondary causes of low HDL-C

1.	Elevated triglycerides.	(Component of metS)
	End stage renal disease	
	Hypothyroidism [also increased total Chol/HDL-C ratio.	
2.	Obesity and Overweight. – [waist measurement] –	(Component of metS)
	[Visceral obesity in particular]	
	For every 3 kg. (7 lbs.) weight loss HDL-C increased 1 mg/dL.	
3.	Prediabetes and overt Type 2 Diabetes Mellitus.	(Component of metS)
4.	Physical inactivity	(lifestyle choice).
5.	Smoking	(lifestyle choice).
6.	Very high carbohydrate intakes > 50–60% of energy	(lifestyle choice).
	[Especially Fructose Containing Soft Drinks.]	
7.	Metabolic Syndrome: As potent a risk factor as smoking.	
8.	Drugs, such as beta-blockers, anabolic steroids, and progestational agents.	

**Table 4:** Beneficial effects of HDL-C

**Figure 1:**
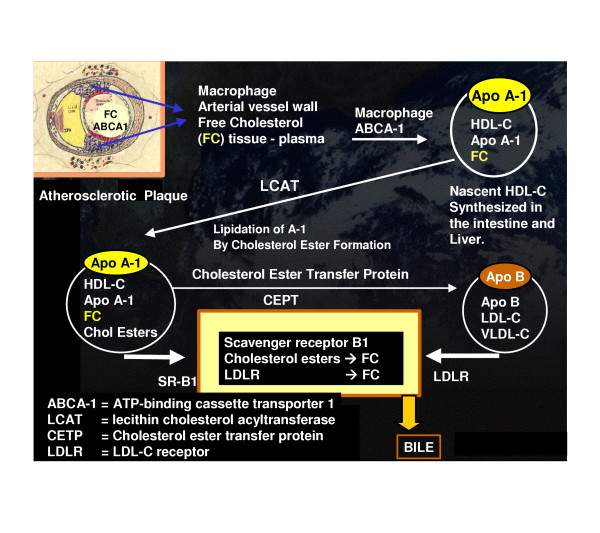
**Reverse Cholesterol Transport. **This figure demonstrates the process of reverse cholesterol transport. It begins in the arterial vessel wall and with the assistance of the ATP binding cassette transporter A-1 (ABCA-1) and in collaboration with the Apo A-1 protein attached to the outer shell of the nascent HDL-C lipoprotein particle free cholesterol is internalized within the HDL-C lipoprotein particle. The enzyme lecithin cholesterol acyltransferase (LCAT) esterifies free cholesterol (FC) via a lipidation process and internalizes it within the HDL-3, which matures to a larger HDL-2 lipoprotein particle. From this point in time the HDL-3 and 2 particles can enter the hepatic cycle via the Scavenger Receptor B-1 and subsequently excreted in the bile. The alternative pathway is for the larger HDL-C apoA-1 lipoprotein particles to undergo a transference of the cholesterol esters through an exchange process with triglycerides via cholesterol ester transfer protein (CETP) to the ApoB-100 lipoprotein particles and enter the liver for further metabolism via the low density lipoprotein receptor (LDLR) to be subsequently excreted in the bile.

**REVERSE CHOLESTEROL TRANSPORT**
Accepts cholesterol from the macrophage and tissues and transports it back to the liver for disposal in the bile (figure 1).
Acts a an apoprotein donor to the other lipoproteins
**ANTIOXIDANT**
Antioxidant activity (through intimal paraoxonase, and redox -sensitive methionine residues of apo A-1)
Increases eNOS and endothelial nitric oxide
**ANTIINFLAMMATORY**
Downregulates adhesion molecule expression on endothelium: (I-CAM, V-CAM and MCP-1)
Inhibits neutrophil degranulation
**ANTITHROMBOTIC**
Antithrombotic activity via its ability to block TxA_2 _and potentiates activity of proteins: C and S.
Stimulates prostacyclin production (antithrombotic and vasodilitory).
**ENDOTHELIAL PROTECTION PROTERTIES**
Acts as an endothelial mitogen and inhibits endothelial cell apoptosis: This would help to decrease the incidence of plaque erosion and promote plaque stabilization
Stimulates endothelial nitric oxide (eNO and its enzyme eNOS) and prostacyclin production with vasodilatation, antioxidant, and anti-inflammatory properties.

HDL-C is synthesized in the intestine and liver and is extremely important in reverse cholesterol transport from the tissues to the liver for disposal. It works in conjunction with the ABCA1 cholesterol transporter within the intimal macrophages (figure 1).

This important dual interaction of HDL-C and the ABCA1 transporter is of great importance and recently we have learned that certain gene polymorphism of ABCA1 transporter may have a profound effect on HDL-C in addition to the well known abnormality of Tangier disease [11]. Probst MC *et al.*, have even set aside a website to list all of the known ABCA1 gene polymorphisms [11].

Oxidative stress and the reductive stress (redox stress) associated with overt T2DM and multiple risk factors associated with accelerated atherosclerosis may result in a damaging effect to HDL-C and interfere with the ABCA1 transporter in reverse cholesterol transport via a mechanism of oxidation and nitration of tyrosine residues on the apo A-1 lipoprotein outer shell of HDL-C lipoprotein [12]. This biochemical alteration of the apo A-1 lipoprotein could disable the process of reverse cholesterol transport and aggravate an underlying isolated low HDL-C level.

Lifestyle changes that are important in raising low HDL-C consist of smoking cessation, weight loss, exercise, and the use of alcohol in moderation.

HDL-C has numerous positive effects on the endothelium and arterial vessel wall, which decrease non-diabetic atherosclerosis and the accelerated atherosclerosis – atheroscleropathy associated with metS and overt T2DM (table 4).

### Emerging novel risk markers of atherosclerosis

NCEP ATP III allows the clinician to factor in the additional risks associated with novel, emerging risk markers such as our patients elevated homocysteine. Other risk markers would be the lipid markers: Elevated triglyceride, remnant lipoproteins, lipoprotein (a), an abnormal TC/HDL-C ratio, small dense LDL particles, HDL subspecies, and apolipoprotein A and B. The non lipid markers would include: An elevated glucose, inflammatory markers (elevated hs-CRP and the emerging importance of the various interleukins and in particular IL-6, which is the driving force behind hs-CRP elevation), coagulation markers (elevated PAI-1, Lp(a), and fibrinogen), the emerging roles of matrix metalloproteinases (MMPs) and of course the established risk marker of an elevated homocysteine. It is interesting to note that Qujeq D et al., noted a negative correlation between total homocysteine and HDL-C levels (p < 0.05, r = 0.93) in a study evaluating 126 patients (67 male and 59 females, aged 29–73 mean of 48.65 +/- 5.79) with unequivocal changes of acute myocardial infarction in the electrocardiogram as compared to 135 normal healthy controls, while noting a positive correlation between total homocysteine and LDL-C levels (p < 0.05, r = 0.98) [13]

The reader may note that the patients' uric acid level was quite elevated prior to his acute coronary event and that this level returned to a very normal level following global risk reduction and aggressive therapy for his multiple risk factors in addition to his isolated low HDL-C. Although not a considered a risk factor or even an emerging, novel risk marker, uric acid may be a quite sensitive marker of underlying redox and oxidative stress. Uric acid levels greater than 4 mg/dL may be considered a red flag in those patients, such as our case report, with high risk for CHD [14].

### The Atherosclerotic Kitchen Sink

When viewing the sources for atherosclerosis it is important to note that there are two routes for accumulation of atherogenic lipoproteins (input and outflow) within the arterial intima and subsequent remodeling of the arterial vessel wall.

The atherosclerosis equation: Lipoprotein Accumulation (retention) in the arterial vessel wall = Lipoprotein in - lipoprotein out. **L-A avw = L-in - L-out.**

L-in, would equal the net lipoproteins derived from the GI tract (absorption) plus that synthesized by the liver. Lipoprotein out is strictly via reverse cholesterol transport to the liver and secretion via bile into the gut. L-in is primarily the beta lipoproteins or apolipoprotein B containing lipoprotein particles, whereas L-out depends primarily on the alpha lipoproteins, apolipoprotein A or HDL-C. The beta lipoproteins are atherogenic and the alpha lipoproteins are antiatherogenic. From this analogy one can see can see why non HDL-C was so important in the recent NCEP ATPIII guidelines: **non HDL-C = total Cholesterol – HDL-C **(reflecting the total atherogenic burden).

This is also why the recent global (52 countries) INTERHART study found the ApoB/ApoA-1 ratio (the ratio of atherogenic lipoproteins to non atherogenic lipoproteins) to be the best predictor of CHD (odds ratio of 3.25 for top verses lowest quintile) as compared to the other eight other risk factors (table 5) [15].

**Table 5:** Nine risk factors account for up to 90 % of MIS worldwide in both sexes, all ages, and in all regions

**RISK FACTOR**	**ODDS RATIO**
Abnormal lipids: ApoB/ApoA-1	3.25
Smoking	2.87
Diabetes	2.37
Hypertension	1.91
Abdominal obesity Reason for such a high OR: This could aggravate smoking, diabetes, hypertension, obesity, alcohol abuse and even nutrition (eating aggressively)	1.12
Psychosocial Factors	2.67
Alcohol use	0.91
Physical Activity	0.86
Consumption of fruits and vegetables	0.70

L-Aavw = ApoB/ApoA-1 ratio of the INTERHART study

L-in, would be comparable to the faucet (GI tract and Liver) delivering the atherogenic apoB lipoproteins. While the kitchen sink would represent the accumulation of atherogenic lipoproteins within the arterial vessel wall or L-Aavw.

**Figure 2:**
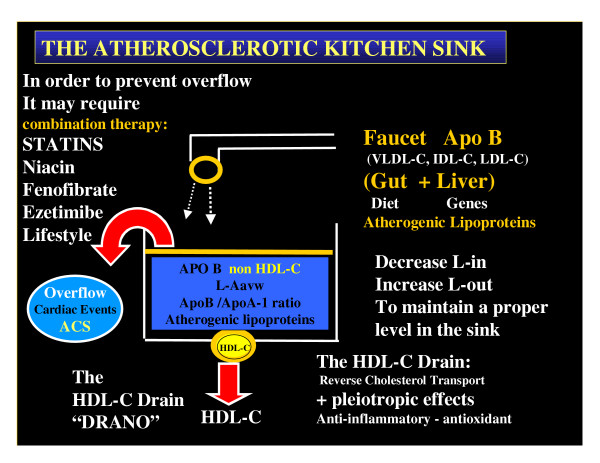
**The Atherosclerotic Kitchen Sink. **This image portrays the importance of the HDL-C drain in maintaining a certain level of atherogenic lipoproteins within the arterial vessel wall to prevent accumulation and the undesirable possibility of an acute event with overflow or acute coronary syndromes. This simple analogy of homeostasis points to an important concept: That being the frequent need for combination therapy in order to control the various components of the atherogenic lipoprofile. Isolated low HDL-C is certainly a red flag regarding the development of atherosclerosis and CHD and additionally the elevation of low HDL-C levels may have a DRANO-LIKE effect to open a clogged drain in an atherosclerotic arterial vessel wall.

In a like manner, the DRAIN would represent L-out or HDL-C or apoA-1 lipoproteins. From this analogy it can easily be seen that if there is inadequate HDL-C or apoA-1 the atherogenic kitchen sink will overflow and result in acute coronary syndromes as happened in our case report (figure 2).

## Conclusion

While the treatment of isolated HDL-C may seem overwhelming at times, it will be rewarding for both the clinician and the patient as demonstrated by the our case study. This patient has done well for seven years and it is anticipated he will continue to do well with his laboratory values now in a sustained, normal physiologic range.

Additional tests by nuclear magnetic resonance spectroscopy (NMR LipoProfile) would assist us in knowing the LDL particle number (LDL-P) and would assist us in even more aggressive therapy. In addition to his current goals he has met, he should have an LDL-P under 1000 micromol/L and small LDL-P under 700 micromol/L.

Even though we have discussed LDL-C from a quantity perspective, due to an isolated low HDL-C, we should additionally be aware that there exists and equally important role for the quality of HDL-C [12]. Recently, the **Apo A-1**_**Milano **_and **Apo A-1**_**Paris **_have resulted in a marked increase in research interest for the HDL-C lipoprotein particle and its future manipulation [16]. In the near future we may be utilizing gene transfer utilizing variations of the Milano and Paris forms, as well as the newer apoA-1 mimetics such as L-4F [17]. Recently there has been increased interest in CETP inhibitors and Phase II studies are underway with torcetrapib and the combination of torcetrapib and atorvastatin [18]. Additional attention to the PPAR agonists and atherosclerosis and the liver X receptor alpha (LXR alpha) agonists is being employed at the present and the positive dual effects on HDL-C and atherosclerosis is being actively investigated. This dual agonism of PPAR alpha, gamma, and possible delta, as well as the dual effects of PPAR alpha and LXR alpha are quite exciting and we will learn a great deal regarding their effects on atherosclerosis and HDL-C in the near future [19].

Recently John Snow, M.D. (1813–1858), a legendary figure in the field of epidemiology, of London, England was honored [20]. He hypothesized that Cholera was transmitted by water rather than miasma (bad air). He suspected the water from the Broad Street pump was the source of the disease and subsequently had the pump handle removed in 1854 (150 years ago) [21].

Could low HDL-C be the "pump handle" of atherosclerosis and CHD?

## List of abbreviations

ABCA-1: ATP binding cassette transporter A-1

BMI: body mass index

CHD: coronary heart disease

CAD: coronary artery disease

hs-CRP: highly sensitive C reactive protein

T2DM: type 2 diabetes mellitus

metS: metabolic syndrome

HDL-C: high density lipoprotein cholesterol

LDL-C: low density lipoprotein cholesterol

LFTs: liver function tests

PCTA: percutaneous transluminal coronary angioplasty

VLDL-C: very low density lipoprotein cholesterol

TC: total cholesterol

TLC: therapeutic lifestyle changes

TPA: tissue plasminogen activator

## Competing interests

The author(s) declare that they have no competing interests.

## Author contribution

MRH conceived the idea to write this manuscript. MRH and SCT wrote, and edited this manuscript together.
